# Eye Inflammation Dependent upon Lesions of Dental Branches of the Fifth Nerve

**Published:** 1888-11

**Authors:** 


### Eye Inflammation Dependent upon Lesions of Dental Branches of the Fifth Nerve.

In the New York *Medical Journal*, April 14, 1888, Dr. F. W. Marlow reports three cases illustrating the dependence of eye-inflammation upon irritative lesions of the dental branches of the fifth nerve. The following is a summary of his paper:

1. The inflammation was most marked at the margin of the cornea. 2. The conjunctiva affection was limited to congestion and slight swelling, there being no discharge of matter. It would thus seem probable that the conjunctival symptoms were simply secondary to the corneal. 3. The corneal affection was quite superficial, but extended over a considerable area. In the first case it much resembled that seen in inherited syphilis. In the last two cases there was a tendency to lose the epithelium. Thus it differed from the ordinary phlyctenular type of keratitis, which tends to be more definitely localized in one or more spots, and to be accompanied by well-marked ulceration and the development of blood-vessels on the cornea.

4. In all the cases there were variations in the condition from time to time, the symptoms never entirely disappearing (with the exception of those of the left eye in one case) in spite of the long-continued use of the usual local and general treatment.

5. When the dental irritation was removed the eye-symptoms disappeared rapidly and completely in each case, and so far without relapse.

There can be little doubt, he says, that the removal of the dental irritation and the disappearance of the eye-symptoms stood to one another in the relation of cause and effect, and he thinks it may fairly be said that the dental irritation aggravated and prolonged the eye-inflammation, if it did not, as indeed seems very probable, constitute the original exciting cause of the trouble. If we admit, he concludes, that the condition of the teeth influenced the eye trouble in any way in these cases, it becomes evident that a dental lesion too slight, or at any rate not of a nature to call attention by symptoms to itself, may yet be sufficient to exert such an influence.

